# Nonbacterial thrombotic endocarditis of mitral valve associated with a lymphoproliferative malignancy: case report and literature review

**DOI:** 10.1186/s40959-024-00226-0

**Published:** 2024-04-19

**Authors:** Fabiana Duarte, Maria Inês Barradas, Ana Raquel Dias, Carlos Faria, Carina Machado, Carolina Pavão

**Affiliations:** 1 Cardiology Department, Hospital of Divino Espírito Santo of Ponta Delgada, Avenida D. Manuel I 9500-370, São Miguel Island, Azores Portugal; 2 Hematology Department, Hospital of Divino Espírito Santo of Ponta Delgada, São Miguel Island, Azores, Portugal; 3 Anatomical Pathology Department, Hospital of Divino Espírito Santo of Ponta Delgada, São Miguel Island, Azores, Portugal

**Keywords:** Non-bacterial thrombotic endocarditis, Non-Hodgkin lymphoma, Raynaud phenomenon, Vascular surgery, Case report

## Abstract

**Background:**

Non-bacterial thrombotic endocarditis (NBTE) is a rare condition marked by sterile vegetations on cardiac valves, often linked to rheumatologic diseases, autoimmune disorders, and advanced solid malignancies. An early diagnosis and treatment of the associated clinical condition are mandatory, although they do not usually eliminate valvular vegetations, making anticoagulation essential to prevent embolic events. Despite variability, the prognosis of NBTE is usually unfavorable due to recurrent embolic events and the severity of the primary condition, typically advanced cancer.

**Case presentation:**

We present a case of a 57 years-old male who presented to the emergency department with a 5-day history of painful bilateral digital edema and color change episodes (from pallor to cyanosis). Physical examination revealed erythrocyanosis in the distal extremities, prompting consideration of secondary Raynaud syndrome.

Despite medical therapy, progressive digital ischemia led to multiple finger amputations. During etiological investigation, anticoagulation tests and autoimmune analysis yielded negative results. A transesophageal echocardiogram was performed, revealing an irregular hyperechogenic mass on the anterior leaflet of the mitral valve without valve dysfunction, and a thoracic computed tomography scan with contrast showed an enlarged right paratracheal lymph node. Histopathological analysis from a transthoracic needle biopsy of the paratracheal lymph node revealed diffuse large B-cell lymphoma. The patient underwent aggressive R-CHOP chemotherapy, achieving a favorable complete response.

**Conclusion:**

This is a particular case involving the occurrence of NBTE and Raynaud phenomenon as the initial paraneoplastic manifestations in a previously healthy young man. Reports of NBTE associated with lymphoproliferative conditions are quite rare, with fewer than ten cases described in the literature. To our knowledge, this is the first case of NBTE specifically associated with diffuse large B-cell lymphoma.

## Introduction

Non-bacterial thrombotic endocarditis (NBTE) is a rare condition characterized by sterile vegetations affecting undamaged heart valves. The mitral valve is the most commonly affected, and these friable aseptic masses are composed of fibrin and platelets, making them prone to recurrent embolic events [1, 2]. NBTE is often diagnosed late and is frequently associated with specific underlying conditions, namely rheumatologic or autoimmune disease, and also malignancies, usually solid adenocarcinomas. Reports of NBTE associated with lymphoproliferative conditions are quite rare [1–3]. A definite diagnosis typically requires histopathological analysis of the removed specimen, but it is usually based on clinical and imaging data, after excluding systemic infection in a patient with underlying risk factors [2–4].

Raynaud´s phenomenon (RP), representing an exaggerated physiological response to specific stressors, is a common finding in clinical practice. However, an abrupt onset of RP with an unfavorable clinical course should prompt further investigation. Although rare, paraneoplastic RP should always be considered, especially in the absence of an identified autoimmune or vascular cause [5, 6]. Here, we report an uncommon case of lymphoproliferative malignancy initially presenting as acute digital ischemia and NBTE. The prognosis is uncertain and largely depends on the clinical response to chemotherapy and the occurrence of possible adverse side effects [7].

## Case presentation

We report a case of a 57-year-old man with a history of smoking habits, and he was not taking any medication. He had no significant medical or family history of autoimmune, rheumatological or neoplastic diseases. The patient presented to the emergency department with a 5-day history of painful and bilateral digital edema, and episodes of color change (from pallor to cyanosis) that worsened in cold environments. A systemic review revealed a 2-month history of unintentional 8 kg weight loss and asthenia. The patient denied fever, night sweats, dyspnea, cough, chest pain, gastrointestinal or urinary symptoms. He also denied recent travels or potential exposure to infectious diseases.

On physical examination, the patient was hemodynamically stable, apyretic and eupneic. Pulmonary and cardiac examination were unremarkable. He had painful erythrocyanosis in the distal extremities of all his fingers, and some scaly lesions were also evident. All pulses were palpable, and Doppler evaluation revealed slow blood flux in the indicator arteries.

He was evaluated by vascular surgery, and the suspicion of Raynaud syndrome arose. The patient was admitted to the Vascular Unit, and triple therapy with vasodilators, including intravenous prostaglandins, endothelin receptor antagonists, and phosphodiesterase inhibitors was initiated. Despite medical therapy, there was no clinical improvement. Digital ischemia progressed, leading to necrosis in the distal and middle phalanges of the left index finger, requiring amputation. Ulcerations on the index and middle fingers of the right hand were also present, requiring surgical debridement.

At that time, the hypothesis of secondary RP was raised, prompting an in-depth etiological investigation. On further questioning, the patient denied previous episodes of biphasic color changes in extremities or paresthesia; no other symptoms were reported, including alopecia, xerostomia, joint tenderness, muscle weakness, migraines, photosensitivity, or any pattern of fever. He denied drug consumption and known vascular or endocrine dysfunction. No history of arthritis, rash, kidney disease, arterial and venous thromboses was reported.

The initial diagnostic workup included vascular mapping by Doppler ultrasound of both upper and lower limbs, that demonstrated an unspecified thickness of the intima-media layer, with arterial patency in all arterial vessels. The blood tests showed a hemoglobin level of 14.7 g/dL (normal range 14.0 – 18.0 g/dL), mild leukocytosis of 12.63 × 10^3^/µL (normal range 4.0 – 11.5 × 10^3^/µL) and thrombocytosis of 609 × 10^3^/µL (normal range 150 – 430 × 10^3^/µL), an elevated sedimentation rate of 36 mm (normal range < 20 mm) and a C-reactive protein level of 2.6 mg/dL (normal range < 0.5 mg/dL). The metabolic panel, coagulation study, and the remaining biochemical and inflammatory markers were within normal limits. Protein electrophoresis and serum immunoglobulins were normal, and immunofixation was negative. Viral serologies for B and C hepatitis, HIV, and syphilis yielded negative results. Serologic testing for rheumatoid factor, anti-dsDNA, antineutrophil cytoplasmic antibody, anti-extractable nuclear antigen and myositis specific antibodies were negative. Complement fractions C3 and C4 were not consumed. Immunoassay for antiphospholipid autoantibodies showed no relevant results in two separated analysis. Cryoglobulins test was also negative. Two sets of blood cultures were collected and were persistently negative. Further serological and molecular testing aimed at identifying specific microorganisms yielded negative results, ruling out blood culture-negative infective endocarditis.

**Fig. 1:**
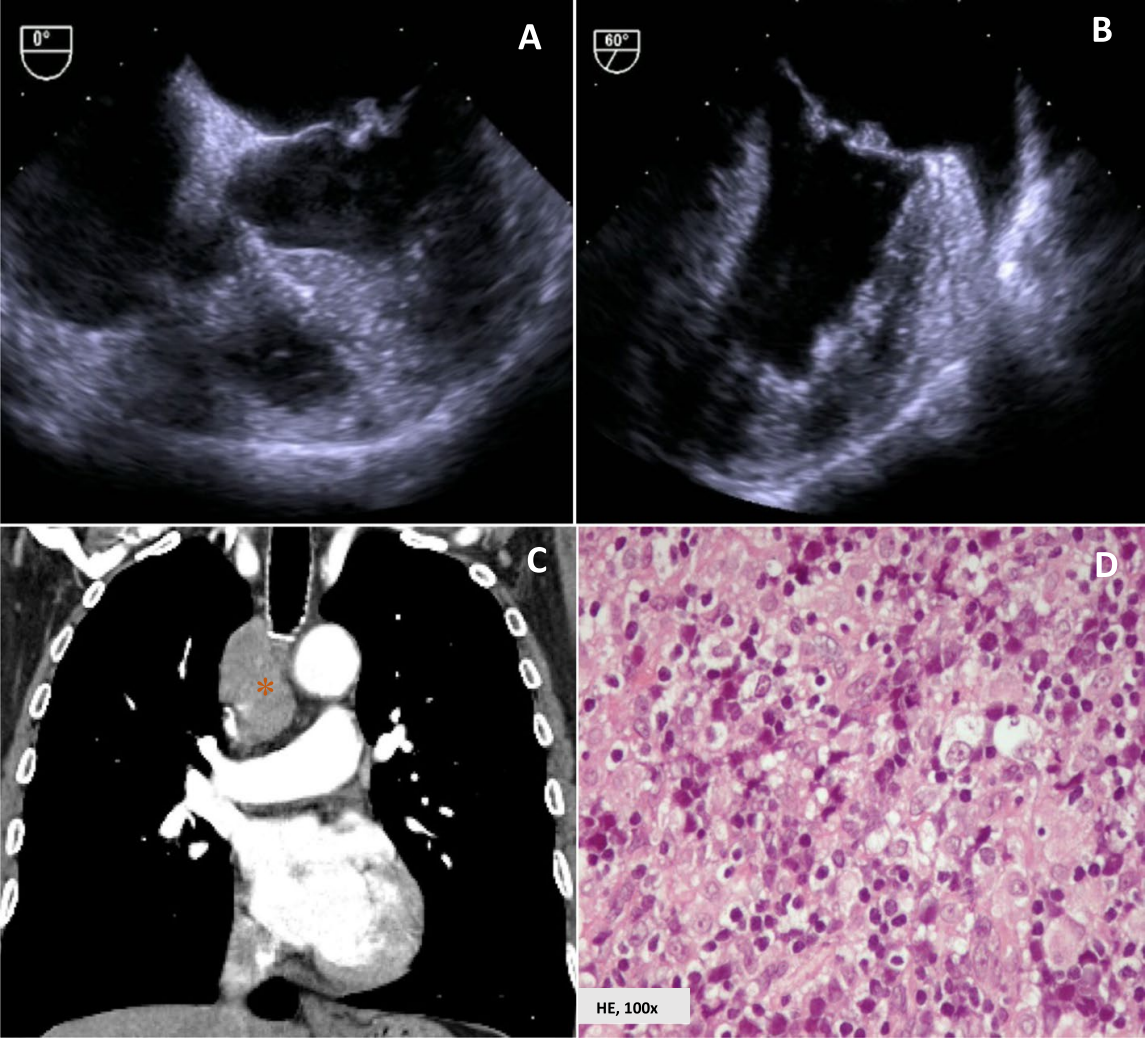
Transesophageal echocardiogram, computed tomography chest scan and histology of the biopsied right paratracheal lymph node mass. Low-middle transesophageal echocardiogram views (Panel** A** and **B**) showing a hyperechoic and irregular mass attached to the atrial aspect of the mitral valve, scallops A1/A2 (7 × 4 mm), without valve obstruction or dysfunction (Panel** A**, apical four-chamber view, 0º and Panel **B**, two-chamber view, 60º). CT chest scan imaging revealed an isolated right paratracheal lymph node mass, with 60 × 37x36 mm (Panel **C**). Hematoxylin and eosin staining of the biopsied lymph node mass showed a lymphoid neoplasia composed of predominantly large cells distributed in a diffuse pattern. The neoplastic cells reveal an eosinophilic cytoplasm with irregular contours and pleomorphic nuclei, showing variable hyperchromasia (Panel **D**)

A transthoracic echocardiogram (TTE), followed by a transesophageal echocardiogram (TOE), showed a hyperechoic and irregular mass attached to the atrial aspect of the mitral valve, scallops A1/A2, measuring 7 × 4 mm, without valve obstruction (Fig. 1).

In this clinical scenario, and considering the negative blood cultures, non-bacterial thrombotic endocarditis was suspected.

Due to the referred changes in white blood cells and platelets count, additional search for possible myeloproliferative neoplasms was performed through peripheral blood molecular testing for *JAK2, MPL* and *CALR* mutations, but the results were negative. Additionally, the patient underwent a thoracic-abdominal-pelvic computed tomography (CT) scan revealing an isolated adenopathic conglomerate in the right lower paratracheal topography, measuring 60 × 37x36 mm (Fig. 1).

At this point, considering the clinical history, laboratory results, and CT scan findings, an immune-mediated or rheumatologic condition was highly unlikely. The patient was discharged with calcium channel blocker, low-dose aspirin, and apixaban, and kept medical follow-up and further etiological investigation.

During the follow-up, finger ulceration progressed, requiring two additional interventions for debridement and amputation of the right index finger. Histologic evaluation of a fragment of a digital artery revealed structural alterations, with intima cell proliferation and intraluminal fibrosis. Due to the poor healing progress of amputated stumps, a complete phalangeal amputation of the right index finger was performed. Additionally, another episode of acute ischemia affecting the left foot had a poor evolution, with irreversible ischemia and areas of necrosis, leading to a transmetatarsal amputation and posterior Chopart amputation.

After multidisciplinary discussion, the patient was scheduled for a transthoracic excisional biopsy of the suspicious mediastinal lymph node through a direct mediastinal approach performed by a thoracic surgeon. The histopathological analysis of the tissue sample was consistent with a diffuse large B cell lymphoma, specifically classified as Germinal Center B-cell type, according to the Hans algorithm [8]. Immunophenotype analysis through immunohistochemistry demonstrated positivity for the following immunomarkers: CD45, CD20, PAX5, BCL6, BCL2 and MYC (Fig. 1). Ki-67 proliferation index was of 50%. Bone marrow biopsy excluded infiltration by lymphoid neoplasm.

The patient had a localized disease with a single lymph node region involved, being included in the Stage I, according to the Ann Arbor staging classification. Based on the revised International Prognostic Index (IPI) and age-adjusted IPI, the patient was included in the low-risk category, presenting a very good prognosis, and he was proposed for a combined chemotherapy regimen with R-CHOP (rituximab plus cyclophosphamide, doxorubicin, vincristine and prednisone). The patient had good clinical response, and there were no further episodes of limb ischemia or necrosis reported. Thoracic CT scan reassessment showed a significant reduction in the adenopathic conglomerate. Echocardiographic reevaluation showed similar findings, with a persistent image of vegetation attached to the mitral valve but without valvular dysfunction. Considering the small size of the vegetation and the absence of valvular dysfunction or embolic events under the initial antithrombotic regiment with direct oral anticoagulant (DOAC), there was no indication for surgery. The patient underwent six cycles of R-CHOP and achieved complete remission, which was confirmed by the absence of metabolic activity in the ^18^F-FDG PET/CT scan.

He kept medical follow-up and showed no signs of disease relapse, secondary tumors, or long-term chemotherapy side effects. RP remained in remission throughout and after the chemotherapy treatment course. Despite the lack of evidence regarding the use of DOACs in such a clinical scenario and considering the favorable clinical evolution and stability of the patient, it was decided to maintain the initially adopted treatment strategy.

## Discussion

Malignancies are associated with a hypercoagulable state and endothelial damage, and NBTE is a rare but possible occurrence in this setting [3, 7]. It is often unrecognized and undiagnosed, with an incidence of approximately 1% retrieved from autopsy examinations, and it remains asymptomatic unless embolization occurs [7]. The incidence of cancer-associated NBTE depends on the type and stage of malignancy. According to some reports, it can affect up to half of patients with solid tumors admitted for echocardiographic screening [2–4]. Frequently associated malignancies include lung, ovarian, pancreatic and esophagogastroduodenal cancers. Reports of NBTE associated with lymphoproliferative conditions are quite rare, with fewer than ten cases described in the literature [2, 7]. For this reason, we conducted a literature review to identify medical reports and collected clinical cases of NBTE associated with hematological diseases in adult patients (Table 1).

**Table 1:** Published clinical cases (including case reports and case series) of non-bacterial thrombotic endocarditis associated with hematological disease in adult patients. Autopsy case reports were not included

**Author, journal, publication year**	**Underlying hematological condition**	**Sex, Age**	**Clinical presentation**	**NBTE diagnosis method(s)**	**Time from neoplasm diagnosis to NBTE**	**Valvular involvement**	**Complications** ^**a**^	**Treatment for NBTE**	**Treatment for HT condition**	**Outcome(s)**
**Ahmed et al. J Oncol Pract. 2018** [9]	T-Cell Large Granular Lymphocytic Leukemia	M, 71 yrs	Petechial rash and mildly decreased neutrophil count	TTE/ TEE	1 year	Anterior mitral valve leaflet; moderate mitral regurgitation	No embolic events; MPGN (immune-mediated sequela of neoplasm)	Long-term anticoagulation using apixaban	No specific treatment was initiated (clinically asymptomatic); MTX followed by CTX for kidney involvement	Improved
**Jeong et al. J Int Korean Med. 2018** [10]	Acute Promyelocytic Leukemia	M, 53 yrs	Fever and abdominal pain	TTE/ TEE	At the same time (during hospital stay)	Aortic valve (right coronary and noncoronary cusp); moderate to severe aortic regurgitation	Systemic embolization	Surgical aortic valve replacement	Arsenic trioxide combined with all-trans retinoic acid CTx	Improved
**Vlismas et al. JACC. Case reports. 2019** [11]	Low grade B-cell lymphoproliferative disorder (probable multiple myeloma)	M, 70 yrs	Chest discomfort	TTE/ TEE	Diagnosis suspected at the same time (during hospital stay); histological confirmation after patient´s death	Quadrivalvular NBTE	Systemic embolization	Anticoagulation therapy (unfractionated heparin)	Not applicable (patient died before definitive diagnosis)	In-hospital death (complete heart block)
**Acuña et al. Mem. Inst. Investig. Cienc. Salud, 2019** [12]	Mixed cellularity classic hodgkin lymphoma	M, 42 yrs	Long-term fever, sweating, asthenia and weight loss	TTE/ TEE	At the same time (during hospital stay)	Mitral valve	No complications	LMWH (prophylactic dose); no long-term anticoagulation	CTx and intravenous steroids	Improved
**Parker et al. Kans. J. Med. 2020** [1]	Acute myelomonocytic Leukemia	F, 29 yrs	Dyspnea on exertion, pallor, fever, chills	On autopsy specimen	1 week later (postmortem diagnosis)	Tricuspid valve	Systemic embolization with multi-organ failure	Not applicable	Cytarabine and daunorubicin CTx	In-hospital death
**Hashimoto et al. Medicina. 2021** [13]	Acute Promyelocytic Leukemia (*post mortem* diagnosis)	F, 45 yrs	Sudden onset of dysarthria, paralysis of right upper and lower extremity and fever	TTE/ TTE; on autopsy specimen	Diagnosis suspected at the same time (during hospital stay); histological confirmation after patient´s death	Aortic valve	Cerebral embolism (initial presentation)	Anticoagulation therapy	Not applicable (patient died before definitive diagnosis)	In-hospital death: intracranial hemorrhage, complication of thrombectomy
**Wang et al. Int J Rheum Dis. 2023** [14]	Burkitt lymphoma (NHL)	F, 20 yrs	Sacroiliac joint pain and fever	TTE/ TEE	At the same time (during hospital stay)	Mitral and aortic valves	No complications	Treatment of underlying neoplastic condition	CTx	Improved

*CTx* chemotherapy, *CTX* cyclophosphamide, *MPGN* Membranoproliferative glomerulonephritis, *MTX* methotrexate, *NHL* Non-Hodgkin lymphoma, *TEE* transoesophageal echocardiogram, *TTE* transthoracic echocardiogram

^a^Related to thromboembolic events and other NBTE or hematological related complications

Regarding cardiac involvement, NBTE usually affects the mitral valve (62%), followed by the aortic valve (up to 24% of cases). Given its friable nature, these sterile vegetations pose a higher risk for fragmentation, leading to recurrent embolic events, up to 82% in malignancy related NBTE [4, 5, 7]. Cardiac valve dysfunction and overt heart failure symptoms are less common [2, 3].

The diagnosis of NBTE relied on the imaging characteristics of the vegetation and the exclusion of an infectious state in a patient with an underlying malignant disease. A comprehensive differential diagnosis involved blood tests and cultures, immunological and serological analysis, ruling out other hypothesis, and the necessity for a histological examination of the specimen [7]. The management of NBTE mainly relies on the treatment of the underlying medical condition in addition to lifelong anticoagulation therapy to prevent systemic embolization.

This is a unique case involving the occurrence of NBTE and RP as the initial paraneoplastic manifestations in a previously healthy young man. The initial clinical presentation included digital ischemia with rapid progression to finger ulceration and necrosis, along with painful and bilateral digital edema, displaying a triphasic discoloration of the affected area. This raised suspicion of secondary RP. An etiological investigation was performed to identify a secondary underlying cause and exclude circulatory, vasospastic, and other conditions that might mimic RP. Through echocardiographic evaluation, an irregular and friable mass attached to the anterior mitral leaflet was discovered, which, given the clinical context, suggested non-bacterial thrombotic endocarditis. The diagnostic workup ultimately led to the diagnosis of a non-Hodgkin lymphoma (NHL), specifically diffuse large B cell lymphoma. The diagnosis of NHL was confirmed, and the risk assessment was stablished based on the Ann Arbor and IPI classification systems ﻿[15]. Then, the patient underwent an aggressive R-CHOP regimen, which produced a favorable and complete response [15].

Notably, our case report presents particular characteristics and important learning points that we would like to highlight:

Firstly, paraneoplastic RP is uncommon, with reported cases typically occurring in the setting of advanced or metastatic disease. Secondary RP is usually more painful and associated with severe ischemic complications, resulting in digital ulcerations and gangrene, sometimes requiring amputation. Albeit rare, when such clinical scenario arises, it should prompt an investigation for an underlying condition, including rheumatic disorders, endocrine conditions, arterial disease and neoplasms, mainly hematologic conditions [6]. Regarding the myriad of possible underlying causes, such clinical scenario represented a diagnostic challenge, culminating in the diagnosis of NBTE and a lymphoproliferative malignancy.

Secondly, unlike previous reports, NBTE was diagnosed early in our case, in the setting of treatment-naïve NHL, and no embolic events occurred during the disease course under systemic anticoagulation [4, 7]. Both aspects represent particular features of our clinical case that contrast with the available evidence. The diagnosis of NBTE was challenging as there are no imaging pathognomonic features, and a constellation of clinical and imaging characteristics occurring in a specific medical context is critical to support the diagnosis, after exclusion of systemic infection [4, 7].

Thirdly, regarding patient follow-up and management therapy, current recommendations support the use of anticoagulant treatment in all patients once the diagnosis of NBTE is established, and its use should be balanced against individual patient´s bleeding risk [16, 17]. Following recommendations, patients should be anticoagulated lifelong with unfractionated heparin or low-molecular-weight heparin, as there are no data regarding the use of DOACs in such clinical scenarios [17–19]. Nevertheless, although heparin is the anticoagulant of choice in NBTE, its use is solely based on limited observational studies [16, 17]. In our clinical case, as the patient remained stable with no additional episodes of systemic embolization, it was decided to maintain the initially adopted treatment strategy with a DOAC (apixaban). This off-label strategy was decided upon by a multidisciplinary team, including Oncology/Hematology, Vascular Surgery and Cardiology, and it was considered reasonable in our middle-age patient without additional risk factors for thromboembolic events, in order to avoid lifelong heparin treatment.

Finally, NBTE is most commonly associated with autoimmune conditions or advanced malignancy, often diagnosed post-mortem. Hematological neoplasms are relatively rare in this context [4, 7]. To the best of our knowledge, our clinical report represents the second case of a NBTE associated with an underlying condition of NHL, being the first case specifically associated with diffuse large B-cell lymphoma [14].

## Data Availability

No datasets were generated or analysed during the current study.

## Abbreviations

CT: Computed Tomography
DOAC: Direct oral anticoagulant
IPI: International Prognostic Index
NBTE: Non-bacterial thrombotic endocarditis
NHL: Non-Hodgkin lymphoma
RP: Raynaud´s phenomenon
TOE: Transesophageal echocardiogram
TTE: Transthoracic echocardiogram
